# Small RNA Expression Profiling by High-Throughput Sequencing: Implications of Enzymatic Manipulation

**DOI:** 10.1155/2012/360358

**Published:** 2012-06-20

**Authors:** Fanglei Zhuang, Ryan T. Fuchs, G. Brett Robb

**Affiliations:** RNA Division, New England Biolabs Inc., 240 County Road, Ipswich, MA 01938, USA

## Abstract

Eukaryotic regulatory small RNAs (sRNAs) play significant roles in many fundamental cellular processes. As such, they have emerged as useful biomarkers for diseases and cell differentiation states. sRNA-based biomarkers outperform traditional messenger RNA-based biomarkers by testing fewer targets with greater accuracy and providing earlier detection for disease states. Therefore, expression profiling of sRNAs is fundamentally important to further advance the understanding of biological processes, as well as diagnosis and treatment of diseases. High-throughput sequencing (HTS) is a powerful approach for both sRNA discovery and expression profiling. Here, we discuss the general considerations for sRNA-based HTS profiling methods from RNA preparation to sequencing library construction, with a focus on the causes of systematic error. By examining the enzymatic manipulation steps of sRNA expression profiling, this paper aims to demystify current HTS-based sRNA profiling approaches and to aid researchers in the informed design and interpretation of profiling experiments.

## 1. Introduction

RNA in eukaryotic cells can be classified into five categories: ribosomal RNAs (rRNA), transfer RNAs (tRNA), messenger RNAs (mRNAs), long noncoding RNAs (lncRNAs), and small RNAs (sRNAs). Over 90% of the total RNA molecules present in a cell are rRNA and tRNA, while sRNAs account for ~1% or less. Eukaryotic regulatory sRNAs are a subset of sRNAs ranging in size from ~20 to 30 nt and include microRNAs (miRNAs), small interfering RNAs (siRNAs), and piwi-interacting RNAs (piRNAs). The functions of these regulatory sRNAs are conserved from plants to animals, which imply their involvement in fundamental cellular processes [1]. Discovery and profiling of these regulatory sRNAs are of primary interest to unravel their regulatory functions.

High-throughput sequencing (HTS) has revolutionized the study of sRNAs by simultaneously accelerating their discovery and revealing their expression patterns. As we have learned from microarray-based sRNA expression profiling [2, 3], key steps in HTS-based profiling workflows warrant careful consideration in order to either avoid introducing systematic error or to guide interpretation of results.

 In this paper, we discuss preparation of sRNAs for profiling by HTS and enzymatic manipulation upstream of sequencing library preparation. The purpose of enzymatic manipulation is either to improve representation and reduce bias or to specifically focus on subsets of sRNAs based on end modifications. Furthermore, we review the activities of the enzymes directly involved in common HTS library preparation methods and discuss their relative strengths and weaknesses to facilitate choosing suitable protocols and interpretation of the results.

## 2. Small RNAs

### 2.1. Classes of Small RNAs and Their Functions

Although small in size, eukaryotic regulatory sRNAs are diverse in their sequences, modifications, biogenesis, expression patterns, and functions [4]. sRNAs have typically been classified based on their transcription origin, processing pathways, interaction with effector proteins, and functionalities.

#### 2.1.1. MicroRNAs (miRNAs)

miRNAs are a class of 21 to 24 nt sRNAs in most eukaryotes that regulate gene expression at the transcriptional or posttranscriptional level [1]. In animals, the mechanism of miRNA biogenesis is evolutionarily conserved and involves sequential endonucleolytic cleavages mediated by the RNase III enzymes Drosha and Dicer. Primary miRNAs (pri-miRNAs) are transcribed by RNA polymerase II and processed into precursor miRNAs (pre-miRNAs) by Drosha in the nucleus. pre-miRNAs are transported to the cytoplasm via exportin-5 [5] and undergo further cleavage by Dicer, resulting in a ~22 nt double-stranded mature miRNA. The mature miRNAs in animals possess a monophosphate at the 5′-termini and a 2′-, 3′-hydroxyl groups at the 3′-termini (Table 1) [6, 7]. Mature miRNAs are bound by Argonaute proteins and incorporated into the RNA-induced silencing complex (RISC), which recognizes target mRNAs through imperfect base pairing and regulates gene expression through destabilization of targeted mRNAs and/or translational repression in the cytoplasm.

Mature miRNAs are present in the cytoplasm, mainly within cytosolic P bodies, stress granules, and in association with polyribosomes [8–12], miRNAs are also found in the nucleus [13–15] and mitochondria [16]. To date, 21643 mature miRNAs from 168 species have been cataloged in miRBase, an online database of miRNAs [17], and the list of miRNAs is expected to increase further according to bioinformatic predictions [18].

miRNAs are now considered to be key regulators of gene expression in higher eukaryotes with estimates that at least 20–30% human protein-coding genes are regulated by miRNAs [19–21]. Regulation of miRNAs plays important roles in diverse biological aspects including developmental timing, haematopoiesis, organogenesis, apoptosis, cell proliferation and tumorgenesis [22]. Defects in miRNA biogenesis or misregulation of miRNA expression can lead to profound developmental defects, diseases, and human malignancies [23].

#### 2.1.2. Small Interfering RNAs (siRNAs)

A second major class of sRNAs is endogenous small interfering RNAs (siRNAs). They are 21 to 23 nt in length and originate from endogenous double-stranded RNAs (dsRNAs) that are either synthesized by RNA-dependent RNA polymerase or that originate from annealed regions within or between endogenous transcripts [24–26]. siRNAs can be generated through Dicer-dependent or independent pathways [27–30]. siRNAs generated by a Dicer-dependent pathway contain a monophosphate group at the 5′-end and siRNAs generated by a Dicer-independent pathway, called secondary siRNAs, possess distinguishing 5′-polyphosphate groups (Table 1) [27–29]. Endogenous siRNAs are involved in degradation mRNAs through perfect base pairing and also guide histones and DNA methylation to confer transcriptional regulation [30].

#### 2.1.3. Piwi-Interacting RNAs (piRNAs)

Piwi-interacting RNAs (piRNAs) are a class of sRNAs that are 26 to 30 nt long and are speculated to be generated from long single-stranded RNA precursors [31–34]. piRNAs are predominantly expressed in germline cell lineages and associate with Piwi proteins, a subfamily of Ago/Piwi proteins, to suppress transposon expression and ensure genome stability [4, 35–37]. Studies of piRNA structure and modification revealed that the 5′-nucleotide of piRNAs is enriched for uridine [35, 38] and their 3′-termini are 2′-O-methylated (Table 1) [39, 40]. The 2′-O-methyl modification of piRNAs may function to prevent exonucleolytic degradation and undesirable terminal uridylation or to facilitate recognition by specific effector proteins [39, 40].

#### 2.1.4. Other Regulatory sRNAs

Besides miRNAs, siRNAs, and piRNAs, many other classes of sRNAs exist and novel classes continue to be discovered in many organisms. For example, tasiRNA, natsiRNA, tncRNA, hcRNA, rasiRNA, scnRNA, and 21U-RNA have been identified recently [4]. Their structures, nucleotide modifications, mechanisms of biogenesis, and functions await complete characterization.

With the employment of new technologies, such as HTS, discovering new classes of sRNAs is more feasible. Understanding their biological roles in various aspects of cellular processes and disease states is an important and exciting scientific frontier.

### 2.2. miRNAs as Biomarkers

Due to their conserved functions in gene regulation, miRNAs have become valuable biomarkers for many diseases and cell differentiation states [41]. For example, during embryonic development in zebrafish, 115 conserved miRNAs show distinct temporal and spatial expression patterns [42]. Compared to traditional mRNA-based gene expression profiling methods, miRNA biomarkers have the advantage of using a smaller number of targets, at the same time providing greater specificity [43]. For example, the expression of 217 human miRNAs in cancer varies dramatically across tumor types and the expression pattern of this small set of miRNAs defines the cancer type better than expression data from 16,000 mRNAs [41]. Furthermore, miRNA expression profiling outperforms mRNA-based expression profiling in formalin-fixed paraffin-embedded (FFPE) tissues, likely because miRNAs are short in length and less susceptible to nucleases [44]. miRNA expression profiles can successfully classify poorly differentiated tumors, whereas mRNA profiles are inaccurate [41, 45]. This enables miRNA biomarkers to be of use as early warning or diagnostic markers for cancer initiation or progression [46]. Due to their regulatory roles in the cell, other sRNAs can be potentially useful biomarkers, for example, six small nuclear RNAs were identified as useful biomarkers for lung cancer detection [47]. To date, sRNA-based biomarkers outperform traditional mRNA biomarkers by testing fewer targets, with increased specificity, better correlation, and earlier detection in disease progression.

### 2.3. miRNA Editing

During their biogenesis, miRNAs can be subjected to various editing events, such as 3′ to 5′ exonucleolytic processing [48, 49], 3′-terminal U, A, and C additions [50, 51], and A-to-I editing by adenosine deaminase [52–54]. The resulting sequence alterations are important posttranscriptional mechanisms to control miRNA processing and expression. To date the most common editing observed in miRNAs is A-to-I editing, which modifies ~16% of human brain pri-miRNAs [55]. The resulting sequence changes from RNA editing can alter secondary structure [52, 56, 57] or affect strand selection within RISC [58, 59]. If the editing occurs in the miRNA “seed” region, which consists of nucleotides 2 to 7 from the 5′-end of mature miRNA sense strand, it can redirect the miRNA to a different mRNA target [52]. miRNA editing does not occur in a random manner and in fact appears to be miRNA gene-, tissue-, and time-specific [60]. One can imagine that similar editing mechanisms might exist for other sRNAs. The biological significance of sRNA editing and the possible impact of its misregulation on diseases remains to be further explored and established. Therefore, expression profiling methods and data analysis protocols that can detect editing events may be important for deciphering disease mechanisms and sRNA functions related to editing.

## 3. Small RNA Extraction, Enrichment, and Preparation

Though focused on HTS-based expression profiling, the methods and principles for preparing samples upstream of sequencing library construction discussed here are also applicable to sample preparation for other RNA expression profiling methods. To profile sRNA expression, it is desirable to avoid introducing systematic error from the sample acquisition, RNA extraction, and preparation. It is also critical that these procedures are thoughtfully considered to ensure reproducibility, valid interpretation, and comparative analysis of profiling results.

### 3.1. Clinical Variables

Clinical research-related sRNA profiling commonly deals with human samples. Age, sex, race, background comorbidity, anesthesia processes, state of consciousness, and circadian rhythms are potentially relevant to miRNA expression profiling [61]. For example, it has been shown that sRNA expression patterns vary according to circadian rhythms *in vivo* and in cell culture [62, 63]. The expression of specific miRNAs varies from different circadian stages in order to regulate the circadian clock through miRNA-mediated translational regulation [64, 65]. Although the impact of these clinical variables on sRNA expression has not been thoroughly investigated, their influence will become clearer as more sRNA expression profiling data accumulates. Hence, it is important to keep these factors the same among samples or to record variations for subsequent data interpretation.

When studying sRNAs from tissues, care must be taken in the tissue processing, which includes tissue procurement, fixation, and embedding. miRNAs appear to be more stable in FFPE tissue than mRNAs, probably due to their small size and reduced likelihood of remaining cross-linked with proteins after proteinase K digestion [66]. Tight correlations of miRNA profiling results were found between fresh tissues versus FFPE tissue, making miRNA profiling an attractive molecular diagnostic target that may be easily incorporated into existing pathology workflows. Expression profiles of many miRNAs are altered relative to stress responses, including nutrient, cell density, and exposure to pathogens [66, 67]. Therefore, attention must be paid to process samples in the same manner in order to control for the triggering additional miRNA responses among samples.

### 3.2. Small RNA Extraction, Enrichment, and Quality Control

sRNAs are often isolated or enriched from extracted total RNA in profiling workflows. Although larger RNAs will eventually be excluded from sRNAs during library preparation, it is critical to maintain the integrity of total RNA to avoid the contamination by degraded large RNAs, especially rRNA. To extract total RNA, routine methods are composed of two steps: deproteinizing RNA in biological samples and precipitation of RNA. Deproteinizing RNA can be achieved by SDS solubilization followed by phenol extraction or TRIzol extraction [68]. It is undesirable to use an SDS solubilization method for samples with large amounts of DNA, such as mammalian cell nuclei as the abundant genomic DNA increases the viscosity of the lysate, which can result in incomplete separation during the phenol extraction. The TRIzol extraction method can achieve separation of protein, DNA, and RNA simultaneously. It is an effective method for isolating total RNA that includes sRNAs from samples. When using other lysis methods, results of expression profiling may be altered under some circumstances due to factors such as the spatial distribution of sRNAs in the cell. Some lysis methods incompletely disrupt cellular membranes and require centrifugation to remove insoluble membranes, which might result in underrepresentation of membrane-associated sRNAs.

Ethanol precipitation of sRNAs is commonly used to recover RNAs from ~20 nt to several kilobases in length. When possible, adding a nucleic acid carrier, such as glycogen, linear polyacrylamide, or tRNA, to the sample or prior to the extraction will increase the yield of extraction and precipitation [68, 69]. Compared to using tRNA as a carrier, glycogen and linear polyacrylamide have the advantage of not interfering in downstream quantitation and enzymatic manipulation. In addition, we recommend centrifuging the precipitation mix at top centrifugation speed (at least 15,000 rcf) for at least 30 minutes to achieve the highest recovery yield of sRNAs.

Many column-based RNA isolation kits are commercially available. A key consideration for choosing whether the kit is suitable for sRNA profiling experiments is the retention of sRNA during extraction. Therefore, attention needs to be paid to select appropriate kits to ensure sRNAs retained with high yield during purification. Many of these kits are designed to isolate RNA based on the nucleic acid affinity to silica-based materials in the presence of chaotropic salts, such as guanidinium isothiocyanate, while proteins and other cellular components pass through. Residual contamination of chaotropic salts through purification is possible and can impair downstream enzymatic reactions. Therefore, thorough column washing is advised.

After RNA extraction, removal of residual genomic DNA using DNase I is necessary to ensure the purity of total RNAs. It is also highly recommended to check the integrity of total RNA before isolating sRNAs. Total RNA quality and quantity can be determined by gel electrophoresis or on a microfluidics-based technology, such as the Agilent 2100 Bioanalyzer (Agilent Technology Inc., Santa Clara, CA, USA) [68]. The integrity of total RNA is commonly assessed by the integrity of two major ribosomal RNAs. The Bioanalyzer is more sensitive in assessing RNA quality than gel electrophoresis, as it detects and shows the peak of sRNA which is sometimes difficult to discern as a band on agarose gels. The following methods can be used to determine RNA concentration with reasonable sensitivity and convenience: gel electrophoresis, UV absorbance determination (e.g., NanoDrop spectrophotometer, Thermo Scientific, Wilmington DE, USA), fluorescent dye binding-based methods (e.g., Qubit Fluorometer, Life Technologies, Carlsbad, CA, USA), and Bioanalyzer analysis.

Though it adds hands-on labor and time, enrichment of sRNAs may be desirable for sRNA library construction because the high abundance of rRNA, tRNA, and mRNA may overwhelm the representation of sRNAs in HTS. sRNAs can be separated from other RNAs using polyacrylamide gel electrophoresis (PAGE). After excising gel pieces in the desired size range, sRNAs can be eluted by crushing and soaking in solution with constant rotation (passive diffusion) or can be more efficiently eluted using an electroelution approach with tubes, such as Mini GeBAflex-tubes (Gene Bio-Application Ltd, Yavne, Israel). Gel extraction allows for the tightest control of RNA size range to be analyzed in downstream procedures. A variation of PAGE fractionation is the FlashPAGE Fractionator (Life Technologies) which is a minielectrophoresis device that runs small scale polyacrylamide tube gels for isolating RNAs below a threshold length [70]. Another approach is to selectively remove the large RNAs by precipitating large size RNAs in the presence of polyethylene glycol (PEG) and salt [71]. After PEG precipitation, the sRNAs remain in supernatant and can be precipitated using ethanol. Similarly, size exclusion using devices such as Centricon centrifugal filter devices (Millipore, Billerica, MA, USA) can be used to separate sRNAs from large RNAs by using columns with a 10,000 Dalton (~30 bp of ssRNA) molecular weight cutoff [72]. Although many means are available for sRNA enrichment, close attention needs to be paid to the size threshold of each method when choosing an appropriate method.

### 3.3. Preparing Small RNAs for Expression Profiling

Due to their different origins and biogenesis pathways, sRNAs differ from each other in their modifications at the 5′- and 3′-termini (Table 1). These modifications can impact the enzymatic steps involved in many sRNA profiling approaches. Awareness of these modifications and how they might impact representation of the sRNAs of interest are important for both the choice of method for sRNA preparation and in the interpretation of sRNA profiling results.

Mature miRNAs and siRNAs from mammals have a monophosphate at their 5′-ends and 2′-, 3′-hydroxyl groups at their 3′-ends [6, 7]. Secondary siRNAs originating from RNA-dependent RNA polymerase activity have a triphosphate at their 5′-ends and 2′-, 3′-hydroxyl groups at their 3′-ends [28, 73, 74]. Sequenced piRNAs show a strong bias for a 5′-uridine [75] and have a 2′-O-methyl modification at their 3′-ends [39, 40, 76]. The 5′-termini of messenger RNAs (mRNAs), viral RNAs, small nuclear RNAs (snRNAs), and heterogeneous nuclear RNAs (hnRNAs) possess methylated cap structures that play roles in their stability and localization [77].

**Figure 1:**
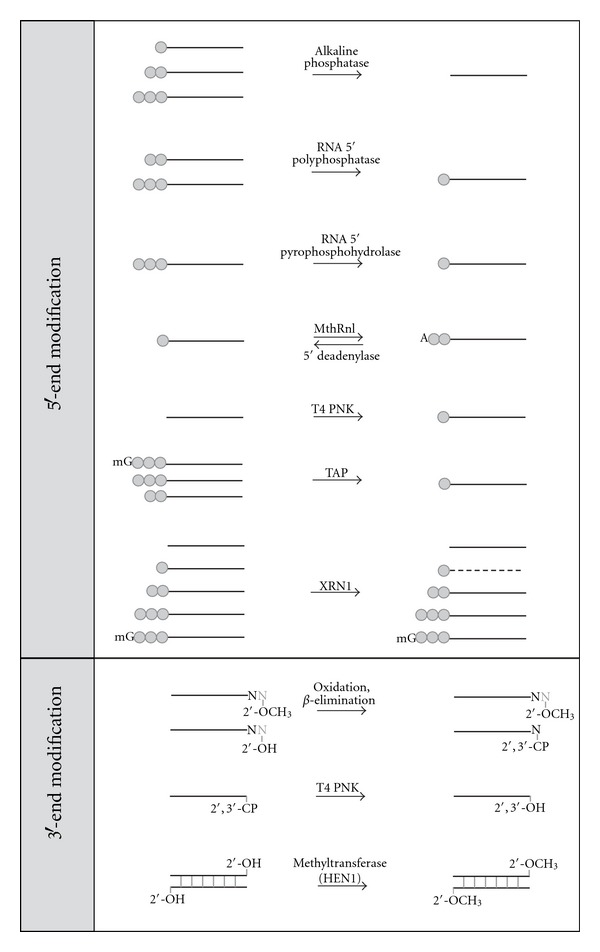
Enzymatic manipulation of RNAs with modifications at their 5′- or 3′-ends. Black lines represent RNA with the left and right ends representing the 5′- and 3′-ends, respectively. One, two, or three grey circles represent mono-, di-, or triphosphate at the 5′-end. “A” and “mG” represent a 3′ to 5′ AMP and cap structure at RNA 5′-end. “2′-OH” or “2′, 3′-OH” represents RNAs with no modification at the 3′-end. “2′-OCH_3_” and “2′, 3′-CP” represent 2′-O-methylation and 2′, 3′-cyclic phosphate at the 3′-end, respectively. Dashed lines represent degraded RNA. The nucleotide “N” in grey color represents the nucleotide removed during the *β*-elimination reaction. MthRnl, TAP, T4 PNK, and XRN1 are the abbreviations of Methanobacterium thermoautotrophicum RNA ligase, tobacco acid pyrophosphatase, T4 polynucleotide kinase, and a 5′-monophosphate-dependent 5′ to 3′exoribonuclease, respectively.

Some sRNA 5′- or 3′-end modifications are not reactive or have reduced reactivity for enzymatic manipulation in expression profiling protocols. For example, the commonly used T4 RNA ligases can efficiently catalyze the formation of a 3′- to 5′-phophodiester bond between a 3′-hydroxyl group and a 5′-phosphate group [78–80]. Therefore, it is sometimes necessary to convert sRNAs of interest to have appropriate and homogenous ends in order to be ligated by T4 RNA ligases with equal and practical efficiency. Alternatively, specific classes of sRNAs as defined by end modifications can be selectively removed or retained within a mix after enzymatic modifications.  Figure 1 summarizes currently available enzymes that can be used to treat and analyze various RNA 5′- and 3′-end modifications.

sRNA 5′-ends can have a 5′-hydroxyl group or contain a mono-, di-, or triphosphate group, or a cap structure. In order to convert sRNAs to have ligatable 5′-monophosphates, a number of enzymes can be utilized, and the choice of enzyme depends on the starting modification and desired enrichment or depletion of different substrates. To capture sRNAs with a 5′-triphosphate, such as secondary siRNAs, the 5′-triphosphate can be removed by alkaline phosphatase to yield a 5′-hydroxyl group. The removal of 5′-phosphate groups to yield a 5′-hydroxyl group has the advantage of preventing RNA self-ligation to form circles and concatemers. This has the net result of improving the yield of properly ligated products when ligating an adapter to the RNA 3′-end [81]. sRNAs with 5′-triphosphate ends can be directly converted into 5′-monophosphate ends using RNA 5′-polyphosphatase, RNA 5′-pyrophosphohydrolase [82], or tobacco acid pyrophosphatase (TAP) [83–85]. The resulting sRNAs with a 5′-monophosphate can be used as a substrate for ligation of an adapter to the 5′-end without further modification. RNAs with a 5′-hydroxyl group, which may result from alkaline phosphatase treatment or chemical synthesis, can be phosphorylated using T4 polynucleotide kinase (T4 PNK) to transfer a monophosphate to the RNA 5′-end.

Instead of 5′-phosphorylated DNA adapters, adenylated DNA adapters are widely ligated to RNA 3′-hydroxyl ends since preadenylation allows for the exclusion of ATP in ligation reactions when using T4 RNA ligases. This leads to decreased formation of self-ligated adapter or adapter concatermers [81, 86]. To synthesize a 5′-adenylated DNA oligo, T4 DNA ligase can be used to adenylate DNA with a 5′-phosphate in the presence of a template DNA that contains at least one unpaired nucleotide opposite to the 5′-phosphate [87]. The thermostable RNA ligase from Methanobacterium thermoautotrophicum (MthRnl) (Figure 1) allows for a much more streamlined and efficient approach to adapter adenylation since single-stranded substrates can be converted with very high efficiency, avoiding the need for gel purification steps [88]. Theoretically, unknown sRNAs adenylated with MthRnl could subsequently be used to directly attach 5′-end adapters using T4 RNA ligase in the absence of ATP, though its use for this purpose has not yet been reported.

It remains to be determined whether there are significant amounts of sRNA species that contain 5′-adenlyated ends *in vivo*. To make these species ligatable, whether naturally occurring or resulting from *in vitro* manipulation, the adenylyl group at an RNA 5′-end can be removed using 5′-deadenylase in a reaction that liberates AMP to yield 5′-monophosphate ends. 5′-deadenylase is also active on 5′-adenylated DNA ends.

TAP hydrolyzes the phosphoric acid anhydride bonds in the triphosphate bridge of the cap structure, releasing the cap nucleoside and generating a 5′-monophosphate terminus on the RNA molecule [89, 90]. RNAs with capped structures include mRNAs, snRNAs, hnRNAs, and some viral sRNAs [77, 91]. For these RNAs, a decapping step is necessary prior to downstream applications such as end mapping and labeling [92, 93], and the same is true for HTS library construction where sequencing of the capped end is desired.

Due to the presence of 5′-monophosphate groups in sRNAs, such as miRNAs and siRNAs, one can selectively degrade these sRNAs using XRN1, a 5′ to 3′exoribonuclease [94]. Degradation of RNA by XRN1 exonuclease is dependent on the presence of a 5′-monophosphate. Therefore, RNAs with a 5′-monophosphate such as miRNAs, siRNAs, or mRNA decapped by TAP can be selectively degraded, while RNA that contains diphosphate, triphosphate, cap structure, or a hydroxyl group at the 5′-end will remain intact. The XRN1 exonuclease therefore has been used to validate the 5′-modification state of RNAs or to enrich RNAs not having a 5′-monophosphate group [95–97].

3′-ends of sRNAs can also be differentially modified during biogenesis. piRNAs, for instance, are methylated at the 2′-position of the 3′-terminal ribose. RNAs with a 3′-end 2′-O-methyl group are ligatable by T4 RNA ligases but with significantly decreased efficiency under standard conditions. Ligation reactions using a mutant variant of T4 RNA ligase 2 (T4 Rnl2), T4 RNA ligase 2 truncated (T4 Rnl2tr), at an optimal PEG concentration can significantly improve 3′-adapter ligation efficiency of RNAs with a 2′-O-methyl 3′-end to a level equivalent to that of unmodified RNAs. As a result, their representation in sRNA quantification experiments will be increased [86]. Conversely, RNAs can be methylated at the 2′-position of their 3′-terminal nucleotides using HEN1 methyltransferase for labeling applications [98]. Theoretically, treatment of sRNA samples with HEN1 would 2′-O-methylate all 3′-ends, potentially equalizing the ligation potential of the entire pool. Commercially available *Arabadopsis* HEN1 is only active on double-stranded sRNAs. HEN1 active on ssRNA* in vitro* is not yet commercially available [99].

To selectively capture sRNAs with a 2′-O-methyl at the 3′-end in HTS libraries, such as piRNAs, RNAs can be treated with oxidation followed by *β*-elimination to convert RNAs with a 2′-hydroxyl group at the 3′-end to form unligatable 2′-, 3′-cyclic phosphate ends that are one base shorter (Figure 1). RNAs with 2′-O-methyl 3′-ends are not converted and then can be selectively captured by ligation [100, 101].

2′-, 3′-cyclic phosphate at RNA 3′-ends can also arise from enzymatic or chemical processing of RNA. In contrast to DNA, the reactive 2′-hydroxyl group on the ribose ring in RNA can promote a hydrophilic attack and breakage of the 5′-, 3′-phosphodiester bond, forming 2′-, 3′-cyclic phosphate ends. RNAs fragmented by treatment with divalent cations or ribozyme-mediated cleavage have a 2′-, 3′-cyclic phosphate at the 3′-end that arise by this mechanism [102]. RNA digested by RNase A, T1, or 1 can have either 2′-, 3′-cyclic phosphate or 2′-hydroxyl, 3′-phosphate ends [103] that are also not substrates for T4 RNA ligases.

Converting the RNA 3′-ends from 2′-, 3′-cyclic phosphate, or 2′-hydroxyl, 3′-phosphate to 2′-, 3′-hydroxyl groups is necessary prior to ligation reactions. This can be achieved by treatment with wild-type T4 PNK with 3′-phosphatase activity, though the pH optimum for the resolution and repair reaction of 2′-, 3′-cyclic phosphate ends is more acidic than for the traditional kinase reaction [102].

In sRNA expression profiling workflows, RNA extraction, enrichment, and enzymatic treatment are potential sources of systematic error upstream of HTS library construction. To ensure representation and accurate quantification of sRNAs, these early steps should be thoughtfully considered and explicitly documented. The full extent of RNA-end modifications is not yet established, and, as novel modifications are discovered, new approaches to prepare RNAs containing these modifications will need to be developed. This will enable realistic interpretation of sRNA profiling data and allow for potential future comparisons.

## 4. High-Throughput Sequencing Library Preparation

HTS approaches have been rapidly adopted for use in sRNA expression profiling. Quantification based on counting-sequenced sRNA species provides a dynamic range that is orders of magnitude greater than traditional microarray approaches, and HTS analyzes orders of magnitude more targets than qPCR. In addition, HTS allows for the identification of new sRNAs with yet-undescribed functions.

### 4.1. Overview of Small RNA HTS Library Construction Methods

HTS sRNA profiling methods generally consist of adding adapters to both ends of sRNAs through various enzymatic reactions and sequencing the resulting sRNA libraries on next-generation sequencers. The idea that HTS can be used for sRNA expression profiling is based on the concept that the relative frequency of sRNAs sequenced correlates to their relative abundance in the sample. However, correlation may be imperfect due to systematic errors in the sRNA preparation protocols. Multiple sources of such bias can be introduced during library preparations including adapter ligation bias from T4 RNA ligases and RNA secondary structures, PCR amplification bias, and bias from sequencing platforms. Building upon the review of RNA modifications and activities of important enzymes used for sRNA profiling in the previous section, we will now examine widely used library construction methods and discuss the potential sources of bias and possible solutions to minimize such bias.

**Figure 2:**
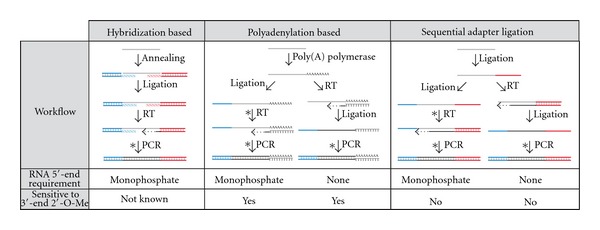
Small RNA high-throughput sequencing library construction methods. The 5′- and 3′-adapters are shown as blue and red lines, respectively. sRNAs are depicted as grey lines. After sRNAs are converted into DNA, the sequences are shown as black lines. The asterisks represent steps suitable for introducing barcodes in each method. The dashed lines with arrows illustrate cDNA synthesis. At the bottom of each schematic diagram, RNA 5′-end requirement and sensitivity to 2′-O-methyl modification at the 3′-end for each method are noted.

Attaching adapters at sRNA 5′- and 3′-ends is required for downstream cDNA synthesis, amplification, and sequencing in HTS.  Figure 2 summarizes commonly used sRNA library construction approaches. Key differences between these methods include the enzymes used and the order of attaching adapters to the 5′- and 3′-ends of sRNAs.

The hybridization-based ligation method (SREK kit for the SOLiD sequencing platform developed by Life Technologies) uses two double stranded adapters that contain degenerate 5′- or 3′-end overhangs. These degenerate overhanging sequences allowing the region to anneal to the unknown sRNA ends. After annealing, the nicks between sRNAs and adapters are sealed using T4 RNA ligases. After ligation, the reaction products are reversed transcribed into cDNA by extending the bottom strand of the 3′-adapter and further amplified using primers annealing to both adapter sequences [104].

A second method utilizes polyadenylation. Multiple A residues are added to the 3′-end using Poly(A) polymerase, creating a 3′ polyA tail [105]. One of two workflows is subsequently followed. In one approach, an RNA adapter is ligated directly to the 5′-end of the tailed sRNAs using a T4 RNA ligase. Adapter-ligated and tailed sRNAs are then reverse transcribed using primers complementary to the homopolymer tail. This workflow requires the sRNA 5′-end to be a 5′-monophosphate in order to be ligated to the 5′-adapter. In an alternate approach, cDNA is synthesized immediately after tailing, and then adapters are ligated to the 3′-end of the nascent cDNA using T4 RNA ligase 1. Since the adapter is ligated to the newly synthesized cDNA, the ligation reaction is independent of the modification state of the RNA 5′-end. After tailing and attaching the adapter, the ligated sRNAs and adapters can be amplified using primers that anneal to the tailed region and 5′-adapter region. A previous study showed that Poly(A) polymerase has a bias toward the last nucleotide at the RNA 3′-end. However, the bias can be greatly minimized by extended reaction time [86]. One significant limitation of Poly(A) based ligation method is that it cannot accurately determine the 3′-end of RNA in the case that RNA ends with the ribonucleotide A. The recent discovery of miRNA editing, particularly at 3′-ends, indicates that the precise determination of RNA 3′-end sequence may be important to understand the biological function of sRNAs. Therefore, quantitative expression profiling approaches may need to take these factors into account.

A third method uses sequential adapter ligations and is widely used for sequencing on the Illumina platform. The method sequentially ligates 3′- and 5′-adapter oligonucleotides directly to the unknown sRNA pools [106]. Similar to the polyadenylation approach, both adapters can be ligated to RNA in sequence followed by cDNA synthesis, or cDNA can be synthesized after 3′-adapter ligation. The later approach therefore does not rely on the 5′-monophosphate of RNA because the 5′-adapter ligates to the newly synthesized cDNA instead of RNA [28]. After the adapter ligations, the reaction products are amplified using primers specifically annealing to the adapter regions.

### 4.2. Library Construction for sRNAs with Modified Ends

Under standard library construction protocols, sRNAs with 2′-O-methyl modifications at their 3′-ends tend to be underrepresented in HTS-based expression profiling experiments due to the effect of the modification on enzymatic reactions. Both polyadenylation and ligation efficiency of RNAs with a 2′-O-methyl group at the 3′-end are less than that of unmodified 3′-ends [86]. Under optimal conditions, the polyadenylation efficiency of RNA with a 2′-hydroxyl group at the 3′-end can approach 100% while RNAs that are 2′-O-methylated at the 3′-end showed a much lower efficiency with significant bias toward the identity of last nucleotide. Therefore, the polyadenylation-based approaches for sRNA profiling are better suited to circumstances in which the modification state of the 3′-ends of the sRNAs of interest is known to be 2′-hydroxyl only, and care should be taken when interpreting HTS datasets that used this library preparation approach. In the ligation-based library construction methods, it is known that the ligation efficiency of 3′-end, 2′-O-methyl modified sRNAs is significantly impaired using T4 RNA ligase 1 (T4 Rnl1) under standard conditions. However, this bias can be significantly reduced using a T4 Rnl2tr with optimized conditions [86].

The reverse transcriptase used for cDNA synthesis is also known to be sensitive to 2′-O-methyl residues in RNA templates [107], which can be another potential source of bias against 2′-O-methyl-modified RNAs. Using either avian myeloblastosis virus (AMV) RT or an excess amount of murine leukemia virus (MLV) RT can greatly minimize the sensitivity of reverse transcriptase to 2′-O-methyl residues in the RNA template [86]. In summary, to efficiently represent sRNAs with 2′-O-methyl groups at their 3′-end in HTS libraries, tailing, adapter ligation, and cDNA synthesis reactions require optimized conditions to be accurately quantitative. 

In order to capture sRNAs with a 5′-triphosphate, such as secondary siRNAs, in HTS libraries, the sRNAs can either be enzymatically treated to convert the 5′-triphosphate to 5′-monophosphate as shown in Figure 1 or can be captured using 5′-monophosphate independent protocols in Figure 2. The 5′-monophosphate-independent protocols perform reverse transcription directly after 3′-adapter ligation or polyadenylation at RNA 3′-ends. The 5′-DNA adapter is then directly ligated to the nascent cDNA using T4 RNA ligase 1. The workflow ignores the modification status of RNA 5′-ends. However, a pitfall for this strategy arises from the ability of reverse transcriptase to add nontemplated nucleotides at the cDNA 3′-end, which will be interpreted as extra nucleotides in the RNA 5′-end after sequencing [108]. Therefore, in sequence datasets prepared using 5′-monophosphate-independent protocols, caution should be used during analysis, particularly with respect to sRNA 5′-ends.

### 4.3. The Use of T4 RNA Ligases in HTS Library Construction

As discussed above, sRNA HTS library construction is achieved through series of enzymatic reactions. T4 RNA ligases are the key enzymes commonly used in all current library construction protocols. Here, we focus on the enzymatic properties of T4 RNA ligases, including T4 Rnl1 and T4 Rnl2, in the context of each library construction protocol.

In the hybridization-based ligation method, ligation is dependent on the annealing of the degenerate region of the adapter to RNAs in the sample. The annealing step itself could potentially introduce bias. We detected significant sequence bias in experiments that used degenerate stem-loop RT primers to sequence random oligonucleotide pools. In the experiments, partially double-stranded stem-loop oligos with 3′-overhanging degenerate regions were designed to hybridize with the 3′-end of sRNAs. HTS data from libraries prepared with this approach showed significant bias toward GC sequences in the hybridizing region. Although the study did not involve use of T4 RNA ligase, it is illustrative of the potential bias in sequence composition when degenerate oligos are used for hybridization [109]. Studies comparing different sequencing preparation methods revealed that sequences of a specific miRNA obtained by the SREK kit displayed a higher nucleotide diversity than those prepared using sequential adapter ligation (Illumina) protocols [110]. This might reflect the occurrence of mispriming in the degenerate region. Thus, care should be taken when drawing conclusions about the identity and precise 3′-ends of sRNAs sequenced using this method.

Another concern in the hybridization-based ligation method arises from the junction substrate specificity of T4 Rnl2. During the annealing process, various types of junctions between RNA and adapter termini can form depending on positioning of the RNA and adapter. Annealing may result in a nick, one or more extra nucleotides flaps at the RNA 5′- or 3′-termini, or gaps. It is known that T4 Rnl2 only promotes the formation of phosphodiester bonds between 3′-hydroxyl ends and 5′-phosphate ends in the nicked arrangement [111]. Annealed RNAs with misaligned termini are not efficiently ligated in this workflow and thus are unlikely to be represented in profiling.

In the sequential adapter ligation protocol, T4 Rnl1 and T4 Rnl2 are used to attach sequence-specific adapters to the 5′- and 3′-end of sRNAs. Although the preferred substrates of both enzymes are RNAs, T4 Rnl1 and T4 Rnl2 are capable of using 5′-phosphate DNA ends as donors, while T4 Rnl1 is also capable of using a DNA acceptor (3′-hydroxyl) [80, 112, 113]. T4 Rnl2 requires at least two ribonucleotide residues at the 3′-end of an otherwise DNA oligonucleotide to have any detectable ligation activity [111]. Therefore, T4 Rnl1 is commonly used for inter- and intramolecular joining of RNA and DNA molecules, and T4 Rnl2 is more commonly used to ligate a nick in dsRNA, splinted RNA ligation, or ligating the 3′-hydroxyl of RNA to the 5′-phosphate of DNA in a double-stranded structure.

T4 Rnl2tr, a C-terminal truncated T4 Rnl2, has desirable features for use in sRNA library construction. The C-terminal domain of T4 Rnl2 is implicated in transferring AMP from ligase to 5′-PO_4_ to form an adenylated RNA intermediate and thus T4 Rnl2tr requires preadenylated donor molecules for ligation [114]. By using T4 Rnl2tr, the ligase-mediated adenylation of RNA 5′-ends is then greatly reduced, which minimizes undesired circularization and concatemerization of RNA and adapters [115]. In ligation reactions using T4 Rnl2tr, the formation of RNA circles and concatemers was found to be significantly reduced by using a DNA adapter with the 5′-end preadenylated and 3′-end blocked using an amino group with no ATP supplied [81, 86, 115]. Further studies on T4 RNA ligases revealed that ligases can deadenylate a 5′-adenylated adapter and utilize the AMP group to adenylate RNAs with a 5′-monophosphate, contributing to concatemerization and circularization of RNAs even in the absence of ATP. The T4 Rnl2tr mutant, K227Q, is an active variant that completely prevents adapter deadenylation activity and thus produces fewer ligation side products [81, 106]. Therefore, the T4 Rnl2tr K227Q mutant is currently the most desirable ligase to ligate a RNA 3′-hydroxyl end to a 5′-adenylated DNA adapter.

A recent study using a pool of synthetic miRNAs showed that the inconsistencies in miRNA quantitation in HTS are mainly derived from the adapter ligation steps [116]. The bias introduced by multiple ligation steps can result in quantification discrepancies as large as three to four orders of magnitude [116, 117]. A number of recent studies have attempted to examine the ligase bias in combination with HTS, in which two adapters were ligated using two different ligases. Therefore, the bias observed reflects a combined bias from two ligation steps using two ligases and two adapters [116, 118]. In these studies, the effect of substrate secondary structure was not exhaustively examined or was not considered. Using a pool of random RNA oligos with full coverage of sequence possibility and studying the ligation bias at the 3′-end ligation step in isolation, a recent study did not detect significant primary sequence bias for RNA substrates by T4 RNA ligases. Instead, the RNA and adapter secondary structure and their cofold structure significantly impact ligation efficiency [109]. Using adapters with random regions showed promise in reducing bias and improving ligation efficiency. Random adapter regions increase the chance that an adapter favorable for ligation is present for each miRNA; however, bioinformatic analysis workflows will need to be adapted to accurately trim randomized adapters from sRNA.

### 4.4. Reverse Transcription

cDNA synthesis by reverse transcriptase (RT) is a common step in all RNA HTS library construction methods. Both fidelity and the ability of RT to synthesize full-length cDNA can potentially impact sRNA profiling by HTS.

In terms of base misincorporation rates, the fidelity of RTs is lower than that of modern proofreading DNA-dependent DNA polymerases used in HTS library construction. While potentially problematic for sRNA variant discovery, we would argue that the contribution of base misincorporation to systematic error in sRNA profiling by HTS is insignificant. The base misincorporation rate of AMV and MLV RTs are ~1/17,000 and ~1/30,000, respectively, as reviewed in [119]. Based on these rates, for cDNAs of 25 nt, we would expect, at worst for these RTs, one molecule in 680 to have one misincorporation. We would expect many fewer 25 nt cDNAs to contain 2 misincorporations if the events are independent and fewer still containing more than 2 misincorporated bases. Short read mapping and counting algorithms for sRNA profiling commonly allow 2 mismatches by default, meaning that the probability of an sRNA being incorrectly counted because of RT base misincorporation is vanishingly small. This suggests that the contribution of RT base misincorporation to systematic error in sRNA profiling by HTS is insignificant.

Insertion and deletion errors by RT are less well characterized than misincorporations, and their impact on sRNA profiling by HTS remains to be determined. Similarly, further elucidation of the causes of RT mutational hotspots [120], if they exist for RTs used in HTS library construction, will be required to determine whether these might affect sRNA profiling.

Untemplated 3′-end nucleotide addition by RT is disadvantageous for protocols synthesizing cDNAs prior to ligation steps. This activity might be problematic for experiments where the precise determination of the sRNA 5′-end is required as discussed in Section 4.2. When precise determination of sRNA 5′-end is required, the protocol of ligating an adapter to the RNA 5′-end followed by cDNA synthesis is preferred. Overall, fidelity of RT in sRNA HTS profiling is likely a small source of systematic error.

RT primer extension of a panel of 5′- and 3′-ligated synthetic miRNAs showed no differences in the yields of cDNA synthesized [116]. These results indicate that template sequence differences (and likely, secondary structure) do not impact the ability of RT to make full-length cDNA for these short templates. Thus, full-length cDNA synthesis by RT of sRNAs is an insignificant source of bias in sRNA HTS library construction [116] and is not likely to impact expression profiling results. The ability of RT to make full-length cDNAs can have more considerable effect on expression profiling when sRNAs are 2′-O-methyl modified as discussed in Section 4.2.

### 4.5. Barcoding

The relatively low number of miRNAs per genome (1520 human miRNAs in miRBase version 18) [17] and their relatively shorter lengths compared to messenger RNAs enable researchers to both discover sRNAs and measure their expression profiles simultaneously. With the increasing throughput accomplished by HTS technologies, one lane of sequencing is already adequate for the identification of novel small RNAs and for their quantification [121]. Considering the cost and time of HTS, multiple samples tagged with distinct sequences, termed barcodes, can be pooled and sequenced in a single lane to lower the cost and increase the throughput of testing numerous biological conditions. Barcodes can be introduced in the adapter for ligation [122, 123], in the RT primer for cDNA synthesis or in primers used for PCR amplification [124–126]. Recent studies showed that barcodes confer bias in HTS-based sRNA expression profiles [127, 128].

Considering the observed bias originating from T4 RNA ligases, introducing barcodes during or prior to any of the ligation steps seems potentially problematic from the viewpoint of sRNA profiling. The different barcode sequences can influence the RNA and adapter cofold structures, likely resulting in barcode-dependent changes in ligation efficiency of sRNAs in the sample. Similarly, changing the adapter sequences can also be expected to change ligation efficiency for specific sRNAs. The net effect of these changes could confound the interpretation of expression profiling results.

Introducing barcodes in the reverse transcription or PCR steps seems less likely to cause biases in estimation of sRNA levels. However, this approach is not without caveats. It is known, for instance, that multiple-template PCR amplification can result in a sequence-dependent amplification bias due to sequence differences [129]. Careful design and testing of barcode placement with the aim of reducing the quantitative bias seems prudent. For example, avoiding introduction of barcodes near primer annealing sites and only including barcoding steps downstream of ligation reactions are potential approaches. The steps we believe to be less problematic for barcoding, in terms of enzyme-based systematic error, are shown in Figure 2 with asterisks in sRNA library construction methods.

From the perspective of RNA ligase-dependent sRNA HTS library construction for expression profiling, it seems clear that library construction protocols, including ligation enzyme reaction conditions and adapter sequences, warrant careful consideration for the interpretation of results. When the libraries are prepared with the same protocol, comparisons of individual miRNA levels between libraries are likely valid and reproducible. Quantification of different sRNAs within a library or quantitation of specific sRNAs between samples prepared with different protocols may be influenced by the protocols themselves. The inclusion of spike-in external standards and careful secondary validation are critical to accurate interpretation of profiling results.

## 5. Alternative sRNA Expression Profiling Methods

Validating expression profiles using alternative methods is essential due to the limitations and systematic error that may exist in any profiling method. Quantitative PCR, Northern Blot hybridizations, and microarrays are widely used methods for sRNA expression profiling [130–132]. The dynamic range of qPCR is higher than HTS, but the dynamic range and sensitivity of microarray and Northern Blot hybridization are much lower. These methods allow the analysis of many fewer sRNAs than HTS and require precise knowledge of sRNA sequence. It is important to note that the measured magnitude of expression levels within samples may differ between HTS and other methods. This is likely because of the protocol differences between the validation approaches and HTS, including the use of enzymes as discussed above.

*In situ* hybridization is also used for sRNA profiling validation. This approach provides the advantages of revealing expression level and localization of sRNA simultaneously but can analyze only a limited number of known targets in a single experiment. This approach has been a useful and powerful tool to provide more insights and focused analyses of individual and limited sets of sRNAs [133, 134].

Other emerging alternative sRNA profiling methods based on electrochemical, bioluminescence, raman signals, and surface plasmon resonance are well discussed in a recent review [132].

Realizing the biases discussed here for current HTS-based profiling, the successful development of an amplification free, direct RNA sequencing platform is particularly attractive to obtain a comprehensive and bias-free profiles of the transcriptome [135], provided that the steps involved in sample preparation are well characterized.

Profiling techniques that provide sensitive detection with large dynamic range but do not require the modification of sRNAs seem ideal. As we look toward the future, new technologies such as nanopores may be able to satisfy some of these criteria. For example, a recent report demonstrated proof of principle for nanopore detection of sRNAs using specific hybridization probes and the viral siRNA binding protein p19 to analyze specific miRNA in a total RNA sample [136]. While this approach is not yet able to determine sRNA sequence, sequence discrimination based on hybridization may be possible in future versions of the technology.

## 6. Summary and Perspective

This paper has discussed many of the sources of inaccuracy and bias that can arise in expression profiling of sRNAs. We focused on the implications of enzymatic manipulation of sRNAs using HTS library construction as an example. Although there are numerous steps where systematic error can be introduced in these workflows, HTS remains the most powerful current method for expression profiling and sRNA discovery. Enzymatic manipulation of nucleic acids in expression profiling will continue to be important, even as hardware platforms change. Thus, it is important that experimental design and interpretation of expression profiling experiments thoughtfully consider the capabilities of enzymes used as tools in order to produce high-quality data sets and to generate valid comparisons between them.

## Figures and Tables

**Table 1:** Classes of small RNAs and their 5′- and 3′-end modifications.

Class	Organism	5′-end modification	3′-end modification
miRNA	Mammals	Monophosphate	2′OH
	Nematodes	Monophosphate	2′OH
	Insects	Monophosphate	2′OH
	Plants	Monophosphate	2′-O-methyl

siRNA	Mammals	Monophosphate	2′OH
	Nematodes	Monophosphate	2′OH
	Insects	Monophosphate	2′-O-methyl
	Plants	Monophosphate	2′-O-methyl

Secondary siRNA	Nematodes	Polyphosphate	2′OH
	Plants	Monophosphate	2′-O-methyl

piRNA	Mammals	Monophosphate	2′-O-methyl
	Nematodes	Monophosphate	2′-O-methyl
	Insects	Monophosphate	2′-O-methyl

For references, see text and [4].

## Abbreviations

sRNAs: Small RNAs
HTS: High-throughput sequencing
rRNA: Ribosomal RNA
tRNA: Transfer RNA
mRNA: Messenger RNA
lncRNA: Long noncoding RNA
miRNA: MicroRNA
siRNA: Small interfering RNA
piRNA: Piwi-interacting RNA
pri-miRNA: Primary miRNA
pre-miRNA: Precursor miRNA
RISC: RNA-induced silencing complex
FFPE tissue: Formalin-fixed paraffin-embedded tissue
snRNA: Small nuclear RNA
hnRNA: Heterogeneous nuclear RNA
TAP: Tobacco acid pyrophosphatase
T4 PNK: T4 polynucleotide kinase
XRN1: A 5′ monophosphate dependent 5′ to 3′exoribonuclease
MthRnl: Methanobacterium thermoautotrophicum RNA ligase
T4 Rnl1: T4 RNA ligase 1
T4 Rnl2: T4 RNA ligase 2
T4 Rnl2tr: T4 RNA ligase 2 truncated
T4 Rnl2trK227Q: T4 RNA ligase 2 truncated with mutation K227Q
AMV RT: Avian myeloblastosis virus reverse transcriptase
MLV RT: Murine leukemia virus reverse transcriptase.
